# On SOCP-based disjunctive cuts for solving a class of integer bilevel nonlinear programs

**DOI:** 10.1007/s10107-023-01965-1

**Published:** 2023-05-27

**Authors:** Elisabeth Gaar, Jon Lee, Ivana Ljubić, Markus Sinnl, Kübra Tanınmış

**Affiliations:** 1 Institute of Production and Logistics Management, Johannes Kepler University Linz, Linz, Austria; 2 JKU Business School, Johannes Kepler University, Linz, Austria; 3 University of Michigan, Ann Arbor, MI USA; 4 ESSEC Business School of Paris, Paris, France

**Keywords:** Bilevel optimization, Disjunctive cuts, Conic optimization, Nonlinear optimization, Branch-and-cut, 90C11, 90C57, 90C30, 65K05

## Abstract

We study a class of integer bilevel programs with second-order cone constraints at the upper-level and a convex-quadratic objective function and linear constraints at the lower-level. We develop disjunctive cuts (DCs) to separate bilevel-infeasible solutions using a second-order-cone-based cut-generating procedure. We propose DC separation strategies and consider several approaches for removing redundant disjunctions and normalization. Using these DCs, we propose a branch-and-cut algorithm for the problem class we study, and a cutting-plane method for the problem variant with only binary variables. We present an extensive computational study on a diverse set of instances, including instances with binary and with integer variables, and instances with a single and with multiple linking constraints. Our computational study demonstrates that the proposed enhancements of our solution approaches are effective for improving the performance. Moreover, both of our approaches outperform a state-of-the-art generic solver for mixed-integer bilevel linear programs that is able to solve a linearized version of our binary instances.

**Supplementary Information:**

The online version contains supplementary material available at 10.1007/s10107-023-01965-1.

## Introduction

Bilevel programs (BPs) are challenging hierarchical optimization problems in which the feasible solutions of the upper-level problem depend on the optimal solution of the lower-level problem. BPs allow us to model two-stage two-player Stackelberg games in which two rational players (often called *leader* and *follower*) compete in a sequential fashion. BPs have applications in many different domains such as machine learning [1], logistics [21], revenue management [36], the energy sector [24, 45] and portfolio optimization [23]. For more details about BPs see, e.g., the book by Dempe and Zemkoho [16] and the recent surveys [7, 31, 48].

In this work, we consider the following integer bilevel nonlinear programs with convex leader and follower objective functions (IBNPs)

1a $$\begin{aligned}&\min ~c'x + d'y \end{aligned}$$

1b $$\begin{aligned} {{\,\mathrm{s.t.}\,}}~&Mx+Ny \ge h \end{aligned}$$

1c $$\begin{aligned}&{\tilde{M}}x+{\tilde{N}}y - {\tilde{h}} \in \mathcal {K} \end{aligned}$$

1d $$\begin{aligned}&y \in \varOmega (x) \end{aligned}$$

1e $$\begin{aligned}&x \in \mathbb Z^{n_1}, \end{aligned}$$

where $\varOmega (x)$ is the set of optimal solutions of the *x*-parametrized so-called *follower* (or *lower-level*) *problem*

2 $$\begin{aligned} \min \left\{ q(y) : Ax+By \ge f,~ y \in \mathcal {Y}, ~y \in \mathbb Z^{n_2} \right\} . \end{aligned}$$

Problem (1) is the so-called *leader* (or *upper-level*) *problem*. The decision variables *x* and *y* are of dimension $n_1$ and $n_2$, respectively, and $n:=n_1+n_2$. Moreover, we have $c \in {\mathbb {R}}^{n_1}$, $d \in {\mathbb {R}}^{n_2}$, $M \in {\mathbb {R}}^{m_1 \times n_1}$, $N \in {\mathbb {R}}^{m_1 \times n_2}$, $h \in {\mathbb {R}}^{ m_1}$, ${\tilde{M}} \in {\mathbb {R}}^{{\tilde{m}}_1 \times n_1}$, ${\tilde{N}} \in {\mathbb {R}}^{{\tilde{m}}_1 \times n_2}$, ${\tilde{h}} \in {\mathbb {R}}^{{\tilde{m}}_1}$, $A \in {\mathbb {Z}}^{m_2 \times n_1}$, $B \in {\mathbb {Z}}^{m_2 \times n_2}$, and $f \in {\mathbb {Z}}^{m_2 }$. We denote by $A^i$, $B^i$ and $f_i$ the *i*-th row of *A*, *B* and *i*-th entry of *f*, respectively. We assume that each $A^i$ and $B^i$ has at least one non-zero entry. The constraints $Ax+By \ge f$ are referred to as *linking constraints*. The constraints (1b)–(1c) are called *coupling constraints*, if they explicitly depend on the follower variables *y*. Furthermore, *q*(*y*) is a convex-quadratic function of the form $q(y) = y'Ry + g'y$ with $R=V'V$ and $V \in {\mathbb {R}}^{n_3 \times n_2}$ with $n_3 \le n_2$, $\mathcal {K}$ is a cross-product of second-order cones, and $\mathcal {Y}$ is a polyhedron. Let *F*(*x*) denote the set of feasible solutions of the follower problem for a given *x*, i.e., $F(x):= \left\{ y \in \mathbb Z^{n_2}: Ax+By \ge f,~ y \in \mathcal {Y} \right\}$. A solution $(x,y) \in \mathbb R^n$ is called *bilevel feasible*, if it satisfies all constraints (1b)–(1e); otherwise it is called *bilevel infeasible*. The IBNP (1) is called *infeasible* if there is no bilevel-feasible solution.

Note that even though the objective function (1a) is linear, we can actually consider any convex objective function which can be represented as a second-order cone constraint and whose optimal value is integer for $(x,y) \in {\mathbb {Z}}^{n}$ (e.g., a convex-quadratic polynomial with integer coefficients). To do so, we can use an epigraph reformulation to transform it into a problem of the form (1).

Our work considers the *optimistic* case of bilevel optimization. This means that whenever there are multiple optimal solutions for the follower problem (2), the one which is best for the leader is chosen, see, e.g., [39]. We note that already mixed-integer bilevel linear programming (MIBLP) is $\Sigma _2^p$-hard [37].

The *value function reformulation* (VFR) of the bilevel program (1) is

3a $$\begin{aligned}&\min ~c'x + d'y \end{aligned}$$

3b $$\begin{aligned} {{\,\mathrm{s.t.}\,}}~&Mx+Ny \ge h \end{aligned}$$

3c $$\begin{aligned}&{\tilde{M}}x+{\tilde{N}}y - {\tilde{h}} \in \mathcal {K} \end{aligned}$$

3d $$\begin{aligned}&Ax+By \ge f \end{aligned}$$

3e $$\begin{aligned}&q(y) \le \Phi (x) \end{aligned}$$

3f $$\begin{aligned}&y \in \mathcal {Y} \end{aligned}$$

3g $$\begin{aligned}&(x,y) \in \mathbb Z^n, \end{aligned}$$

where the so-called *value function* $\Phi (x)$ of the *follower problem*

4 $$\begin{aligned} \Phi (x):= \textrm{min}\left\{ q(y): y \in F(x) \right\} \end{aligned}$$

is typically non-convex and non-continuous. Note that in the optimistic bilevel setting, the VFR is equivalent to the original bilevel program (1). The *high-point relaxation* (HPR) is obtained when dropping (3e), i.e., the optimality condition of *y* for the follower problem, from the VFR  (3). We denote the continuous relaxation (i.e., replacing the integer constraint (3g) with the corresponding variable bound constraints) of the HPR as $\overline{\text{ HPR }}$.

### Contribution and outline

Since the seminal work of Balas [5], and more intensively in the past three decades, disjunctive cuts (DCs) have been successfully exploited for solving mixed-integer (nonlinear) programs (MI(N)LPs) [6]. While there is a plethora of work on using DCs for MINLPs [8], we are not aware of any previous applications of DCs for solving IBNPs. In this work, we demonstrate how DCs can be used within a branch-and-cut (B &C) algorithm to solve (1). This is the first time that DCs are used to separate bilevel-infeasible solutions, using a cut-generating procedure based on second-order cone programming (SOCP). Moreover, we also show that our DCs can be used in a finitely-convergent cutting-plane procedure for 0-1 IBNPs, where the HPR is solved to optimality before separating bilevel-infeasible solutions.

In our preliminary study [22], we described the methodological foundations of our approach, based on the assumption of having a single linking constraint in the follower problem. In this paper, we generalize these results for multiple linking constraints (leading to a cut-generating SOCP with multiple disjunctions). We additionally compare DCs derived from non-optimal versus optimal follower solutions, and show that they are not dominating one another. Moreover, we discuss efficient methods for eliminating redundant disjunctions and normalization procedures for solving the cut-generating SOCP.

Our computational study, which is considerably extended compared to [22], is conducted on instances in which the follower minimizes a convex-quadratic objective function, subject to covering constraints linked with the leader. We consider instances with a single and with multiple linking constraints, and instances with only binary and with integer variables. We demonstrate that the proposed enhancements of our solution algorithms improve their performance. Furthermore, we compare our B &C and cutting-plane approaches with a state-of-the-art solver for MIBLPs (which can solve our binary instances after applying linearization in a McCormick fashion) and we show that the latter one is outperformed by our new DC-based approaches.

Our article is organized as follows. In the remainder of this section, we discuss previous and related work. In Sect. 2 we describe the derivation of the DCs. Section 3 contains a discussion of computational methodology which allows a successful use of our DCs. In particular, in Sect. 3.1 we demonstrate that our DCs cut off bilevel-infeasible solutions. In Sect. 3.2, we demonstrate that cuts derived from optimal follower solutions need not dominate cuts derived from non-optimal follower solutions. In Sect. 3.3, we describe two different separation strategies, in Sect. 3.4, we discuss several approaches to remove redundant disjunctions, and in Sect. 3.5, we address normalization. We present a B &C algorithm for solving (1) in Sect. 4.1 and a cutting-plane algorithm for 0-1 IBNPs in Sect. 4.2. In Sect. 5, we present a computational study, together with some implementation details. In Sect. 6, we conclude with an outlook to further work.

### Literature overview

In recent years, there has been considerable research interest on BPs. When it comes to solution approaches, a distinction between problems with convex and non-convex follower problem can be made. For BPs with a convex follower problem, single-level reformulation techniques based on, e.g., Karush-Kuhn-Tucker optimality conditions or strong duality (see, e.g., [12, 30, 32]) can be used. For MIBLPs with integrality restrictions on (some of) the follower variables, state-of-the-art methods are usually based on B &C (see, e.g., [18, 19, 49]). Other interesting concepts are based on multi-branching, see [50, 52].

Considerably fewer results are available for nonlinear BPs, and in particular with integrality restrictions in the follower problem. In [41], Mitsos et al. propose a general approach for non-convex follower problems which solves nonlinear optimization problems to compute upper and lower bounds in an iterative fashion. In a series of papers on the so-called *branch-and-sandwich* approach, tightened bounds on the optimal value function and on the leader objective-function value are calculated [33–35]. A solution algorithm for mixed-IBNPs proposed in [40] by Lozano and Smith approximates the value function by dynamically inserting additional variables and big-M type constraints. Recently, Kleinert et al. [30] considered BPs with a mixed-integer convex-quadratic leader and a continuous convex-quadratic follower problem. The method is based on outer approximation after the problem is reformulated into a single-level one using strong duality and convexification. In [11], Byeon and Van Hentenryck develop a solution algorithm for BPs, where the leader problem can be modeled as a mixed-integer SOCP and the follower problem can be modeled as a SOCP. The algorithm is based on a dedicated Benders decomposition method. In [51], Weninger et al. propose a methodology that can tackle any kind of MINLP for the leader which can be handled by an off-the-shelf solver. The mixed-integer follower problem has to be convex, bounded, and satisfy Slater’s condition for the continuous variables. This exact method is derived from a previous approach proposed in [53] by Yue et al. for finding feasible solutions. For a more detailed overview of the recent literature on computational bilevel programming we refer to [13, 31, 48].

The only existing application of DCs in the context of bilevel *linear* programming is by Audet et al., [4] who derive DCs from LP-complementarity conditions. In [25], Júdice et al. exploit a similar idea for solving mathematical programs with equilibrium constraints.

DCs are frequently used for solving MINLPs (see [6] and the many references therein, and for example [15, 17, 46, 47]). Concerning the existing literature that includes (computational) studies on DCs for mixed-integer SOCPs, we refer the reader to [2, 3, 14, 27–29, 38, 42] and further references therein.

## Disjunctive cut methodology

The aim of this section is to derive DCs for the bilevel program (1) with the help of SOCP; so we want to derive DCs that separate bilevel-infeasible solutions from the convex hull of bilevel-feasible solutions. Toward this end, we assume throughout this section that we have a second-order conic convex set $\mathcal {P}$, such that $\mathcal {P}$ is a subset of the set of feasible solutions of the $\overline{\text{ HPR }}$. This implies that $\mathcal {P}$ fulfills (3b), (3c), (3d) and (3f) and potentially already some DCs. Moreover, we assume that $(x^*,y^*)$ is a bilevel-infeasible point in $\mathcal {P}$.

### Preliminaries

Our general assumptions regarding the structure of the IBNP are given below.

#### Assumption 1

All variables are bounded in the HPR, and $\mathcal {Y}$ is bounded.

We note that in a bilevel-context already for the linear case of MIBLPs, unboundedness of the $\overline{\text{ HPR }}$ does not imply anything for the original problem, all three options (infeasible, unbounded, and existence of an optimum) are possible. For more details see, e.g., [19].

#### Assumption 2

$\overline{\text{ HPR }}$ has a feasible solution satisfying its nonlinear constraint (3c) strictly, and its dual has a feasible solution.

Assumption 2 ensures that we have strong duality for $\overline{\text{ HPR }}$ and its dual, and so we can solve the $\overline{\text{ HPR }}$ (potentially with added cuts) to arbitrary accuracy.

### Deriving disjunctive cuts

To derive DCs, we first examine bilevel-feasible sets. It is easy to see, and also follows from results of Fischetti et al. [18], that for any ${{\hat{y}}} \in \mathcal {Y} \cap {\mathbb {Z}}^{n_2}$ the set

$$\begin{aligned} S({{\hat{y}}}):= \{ (x,y): Ax \ge f - B{\hat{y}},~ q(y) > q({\hat{y}}) \} \end{aligned}$$

does not contain any bilevel-feasible solution, as for any $(x,y) \in S({{\hat{y}}})$ clearly ${{\hat{y}}}$ is a better follower solution for *x* than *y*. Furthermore, due to the integrality of our variables and of *A* and *B*, the extended set

$$\begin{aligned} S^+({{\hat{y}}}):= \{ (x,y): Ax \ge f - B{\hat{y}}-1,~ q(y) \ge q({\hat{y}}) \} \end{aligned}$$

does not contain any bilevel-feasible solution in its interior, because any bilevel-feasible solution in the interior of $S^+({{\hat{y}}})$ is in $S({{\hat{y}}})$. Based on this observation, intersection cuts have been derived in [18]. However, $S^+({{\hat{y}}})$ is not convex in our case, so we turn our attention to DCs. For any ${{\hat{y}}} \in \mathcal {Y} \cap {\mathbb {Z}}^{n_2}$, any bilevel-feasible solution is in the disjunction $\mathcal {D}_0({\hat{y}}) \vee \mathcal {D}_1({\hat{y}}) \vee \ldots \vee \mathcal {D}_{m_2}({\hat{y}})$, where

$$\begin{aligned} \mathcal {D}_0({\hat{y}}) : q(y) \le q({\hat{y}}) \quad \text { and } \quad \mathcal {D}_i({\hat{y}}) : A^i x \le f_i - B^i {\hat{y}} - 1, \quad i=1,\ldots ,m_2. \end{aligned}$$

To find a DC, we want to generate valid linear inequalities for

$$\begin{aligned} \left\{ (x,y)\in \mathcal {P}: \mathcal {D}_0({\hat{y}})\right\} \vee \bigvee _{i=1}^{m_2} \left\{ (x,y)\in \mathcal {P}: \mathcal {D}_i({\hat{y}})\right\} , \end{aligned}$$

so in other words we want to find a valid linear inequality that separates the bilevel-infeasible solution $(x^*,y^*)$ from the convex hull of the union of multiple disjunctions, namely from

5 $$\begin{aligned} \mathcal {D}({\hat{y}},\mathcal {P}):= {{\,\textrm{conv}\,}}\left( \left\{ (x,y)\in \mathcal {P}: \mathcal {D}_0({\hat{y}})\right\} \cup \left( \bigcup _{i=1}^{m_2} \left\{ (x,y)\in \mathcal {P}: \mathcal {D}_i({\hat{y}})\right\} \right) \right) . \end{aligned}$$

Toward this end, we first derive a formulation of $\mathcal {P}$. If we have already generated some DCs of the form $\alpha 'x +\beta 'y \ge \tau$, then we group them as $\mathcal {A}x + \mathcal {B} y \ge \mathcal {T}$. We take these cuts, together with $Mx+Ny \ge h$ and $Ax+By \ge f$ and also $y \in \mathcal {Y}$, which can be represented as $\mathcal {C} y \ge \mathcal {U}$, and we bundle them all together as

6 $$\begin{aligned}&{\bar{M}}x + {\bar{N}}y \ge {\bar{h}}, \end{aligned}$$

such that $\mathcal {P}$ is represented by (6) and (3c), and where

$$\begin{aligned} {{\bar{M}}} := \begin{pmatrix} M \\ A \\ \mathcal {A} \\ 0 \end{pmatrix} , \qquad {{\bar{N}}} := \begin{pmatrix} N \\ B \\ \mathcal {B} \\ \mathcal {C} \end{pmatrix} , \qquad {{\bar{h}}} := \begin{pmatrix} h \\ f \\ \mathcal {T} \\ \mathcal {U} \end{pmatrix} . \end{aligned}$$

The representation of $\mathcal {D}_i({\hat{y}})$, $i=1,\dots ,m_2$, is straightforward. It is convenient to write $\mathcal {D}_0({\hat{y}})$ in SOCP-form using a standard technique. Indeed, $\mathcal {D}_0({\hat{y}})$ is equivalent to the standard second-order (Lorentz) cone constraint $z^0 \ge \left\| (z^1,z^2) \right\|$ with

$$\begin{aligned} z^0 := \frac{1-\left( g'y- q({\hat{y}}) \right) }{2}, \qquad z^1 := Vy, \qquad z^2: = \frac{1+\left( g'y- q({\hat{y}})\right) }{2}. \end{aligned}$$

Because $z^0$, $z^1$ and $z^2$ are linear in *y*, we can as well write it in the form

7 $$\begin{aligned}&{\tilde{D}}y - {\tilde{c}} \in \mathcal {Q}, \end{aligned}$$

where $\mathcal {Q}$ denotes a standard second-order cone, which is self dual, and

$$\begin{aligned} {\tilde{D}}:= \left( \begin{array}{c} -\frac{1}{2}g' \\ V \\ \frac{1}{2}g' \end{array} \right) \qquad \text { and } \qquad {\tilde{c}}:= \left( \begin{array}{c} \frac{-1-q({\hat{y}})}{2} \\ 0 \\ \frac{-1+q({\hat{y}})}{2} \end{array} \right) . \end{aligned}$$

We employ a vector $\sigma$ of dual multipliers for the linear constraints representing $\mathcal {D}_1({\hat{y}}),\ldots ,\mathcal {D}_{m_2}({\hat{y}})$. Moreover, we employ a vector $\rho \in \mathcal {Q^*}$ of dual multipliers for the constraint (7), representing $\mathcal {D}_0({\hat{y}})$. Furthermore, we employ vectors ${\bar{\pi }}_i$, $i=0,\ldots ,m_2$, of dual multipliers for the constraints (6), and we employ vectors $\tilde{\pi }_i$, $i=0,\ldots ,m_2$, of dual multipliers for the constraints (3c), both together representing $\mathcal {P}$. Then every $(\alpha ,\beta ,\tau )$ corresponding to a valid linear inequality $\alpha 'x +\beta 'y \ge \tau$ for $\mathcal {D}({\hat{y}},\mathcal {P})$ corresponds to a solution of

8a $$\begin{aligned}&\alpha ' = {\bar{\pi }}_i'{\bar{M}} + \tilde{\pi }_i'{\tilde{M}} +\sigma _i A^i{} & {} \forall i =1,\ldots , m_2 \end{aligned}$$

8b $$\begin{aligned}&\alpha ' = {\bar{\pi }}_0'{\bar{M}} + \tilde{\pi }_0'{\tilde{M}} \end{aligned}$$

8c $$\begin{aligned}&\beta ' = {\bar{\pi }}_i'{\bar{N}} + \tilde{\pi }_i'{\tilde{N}}{} & {} \forall i =1,\ldots , m_2 \end{aligned}$$

8d $$\begin{aligned}&\beta ' = {\bar{\pi }}_0'{\bar{N}} + \tilde{\pi }_0'{\tilde{N}} + \rho ' {\tilde{D}} \end{aligned}$$

8e $$\begin{aligned}&\tau \le {\bar{\pi }}_i'{\bar{h}} + \tilde{\pi }_i'{\tilde{h}} + \sigma _i(f_i-1-B^i{\hat{y}}){} & {} \forall i =1,\ldots , m_2 \end{aligned}$$

8f $$\begin{aligned}&\tau \le {\bar{\pi }}_0'{\bar{h}} + \tilde{\pi }_0'{\tilde{h}} + \rho ' {\tilde{c}} \end{aligned}$$

8g $$\begin{aligned}&\sigma \le 0,~ \rho \in \mathcal {Q^*},~{\bar{\pi }}_i \ge 0,~ \tilde{\pi }_i \in \mathcal {K^*}{} & {} \forall i=0,\ldots ,m_2, \end{aligned}$$

where $\mathcal {K}^*$ and $\mathcal {Q^*}$ are the dual cones of $\mathcal {K}$ and $\mathcal {Q}$, respectively (see, e.g., Balas [6, Theorem 1.2]).

To attempt to generate a valid inequality for $\mathcal {D}({\hat{y}},\mathcal {P})$ that is violated by the bilevel-infeasible solution $(x^*,y^*)$, we solve

$$\begin{aligned}&\max \ \tau - \alpha 'x^* -\beta 'y^* \qquad \qquad (CG-SOCP)\\ {{\,\mathrm{s.t.}\,}}&~(8a)\text{-- }(8g). \end{aligned}$$

A positive objective value for a feasible $(\alpha ,\beta ,\tau )$ corresponds to a valid linear inequality $\alpha 'x +\beta 'y \ge \tau$ for $\mathcal {D}({\hat{y}},\mathcal {P})$ violated by $(x^*,y^*)$, i.e., the inequality gives a DC separating $(x^*,y^*)$ from $\mathcal {D}({\hat{y}},\mathcal {P})$. Finally, we need to deal with the fact that the feasible region of (CG-SOCP) is a cone. We will take care of this in the usual manner, by including a normalization constraint; see Sect. 3.5.

## Computational methodology for our disjunctive cuts

In this section we discuss theory and methodology of our proposed DCs.

### Separation theory

To be able to derive DCs we make the following additional assumption.

#### Assumption 3

The dual of (CG-SOCP) has a feasible solution in its interior, and we have an exact solver for (CG-SOCP).

The following theorem allows us to use DCs in our solution methods.

#### Theorem 1

Let $\mathcal {P}$ be a second-order conic convex set, such that $\mathcal {P}$ is a subset of the set of feasible solutions of the $\overline{\text{ HPR }}$. Let $(x^*,y^*)$ be a bilevel-infeasible extreme point of $\mathcal {P}$. If ${{\hat{y}}}$ is a feasible solution to the follower problem for $x=x^*$, i.e., ${{\hat{y}}} \in F(x^*)$, such that $q({\hat{y}}) < q(y^*)$, then there is a DC that separates $(x^*,y^*)$ from $\mathcal {D}({\hat{y}},\mathcal {P})$ and it can be obtained by solving (CG-SOCP).

#### Proof

Assume that there is no cut that separates $(x^*,y^*)$ from $\mathcal {D}({\hat{y}},\mathcal {P})$, then $(x^*,y^*)$ is in $\mathcal {D}({\hat{y}},\mathcal {P})$. However, due to the definition of ${\hat{y}}$, the point $(x^*,y^*)$ does not fulfill $\mathcal {D}_i({\hat{y}})$ for any $i = 0, \dots , m_2$. Therefore, in order to be in $\mathcal {D}({\hat{y}},\mathcal {P})$, the point $(x^*,y^*)$ must be a convex combination of some points $(x_i,y_i)$ for $i = 0, \dots , m_2$, such that each $(x_i,y_i)$ is in $\mathcal {P}\cap \mathcal {D}_i({\hat{y}})$, and such that at least two coefficients of the convex combination are larger than zero. This is not possible due to the fact that $(x^*,y^*)$ is an extreme point of $\mathcal {P}$. Thus, there is a cut that separates $(x^*,y^*)$ from $\mathcal {D}({\hat{y}},\mathcal {P})$. By construction of (CG-SOCP) and due to Assumption 3, we can use (CG-SOCP) to find it. $\square$

Note that there are two reasons why a feasible $\overline{\text{ HPR }}$ solution $(x^*,y^*)$ is bilevel infeasible: it is not integer or $y^*$ is not an optimal follower solution for $x^*$. Thus, in the case that $(x^*,y^*)$ is integer, there is a better follower solution ${\tilde{y}}$ for $x^*$. Then Theorem 1 with ${\hat{y}} = {\tilde{y}}$ implies that $(x^*,y^*)$ can be separated from $\mathcal {D}({\hat{y}},\mathcal {P})$. We present solution methods based on this observation in Sect. 4. In the case that $(x^*,y^*)$ does not fulfill the integer constraints (3g), we distinguish the following situations:

- $F(x^*)\ne \emptyset$ and there is a better feasible follower solution ${\tilde{y}}$ for $x^*$, so one could still use a DC to eliminate $(x^*,y^*)$ due to Theorem 1 with ${\hat{y}} = {\tilde{y}}$.
- $F(x^*)\ne \emptyset$ and all ${\tilde{y}}$ that are feasible for the follower problem $\Phi (x^*)$ have worse (or same) follower objective-function value than $y^*$, so there is no ${\tilde{y}}$ that we can choose as ${\hat{y}}$ in Theorem 1.
- $F(x^*)=\emptyset$, i.e., the follower problem is infeasible for the given fractional point $(x^*,y^*)$.

In the latter two cases, the point $(x^*,y^*)$ cannot be cut off using a DC, however we will see below that such points can be discarded using standard integer-programming techniques. Hence, this potential failure to separate a $(x^*,y^*)$ not fulfilling the integer constraints does not affect our solution algorithms.

### Choosing the point ${{\hat{y}}}$ to separate

For a given DC $\alpha 'x +\beta ' y \ge \tau$, we say that the DC is *dominated* if there exists another DC $\bar{\alpha }'x +\bar{\beta }' y \ge \bar{\tau }$ such that $\{ (x,y) \in {\mathcal {P}}: \bar{\alpha }'x +\bar{\beta }' y \ge \bar{\tau }\} \subset \{ (x,y) \in {\mathcal {P}}: \alpha 'x + \beta ' y \ge \tau \}$, otherwise the DC is called *non-dominated*. If $\{ (x,y) \in {\mathcal {P}}: \alpha 'x + \beta ' y \ge \tau \} \subset \{ (x,y) \in {\mathcal {P}}: \bar{\alpha }'x +\bar{\beta }' y \ge \bar{\tau }\}$, we say that the DC $\alpha 'x +\beta ' y \ge \tau$ is *dominating* the DC $\bar{\alpha }'x +\bar{\beta }' y \ge \bar{\tau }$, otherwise the DC $\alpha 'x +\beta ' y \ge \tau$ is *not dominating* the DC $\bar{\alpha }'x +\bar{\beta }' y \ge \bar{\tau }$. The following result establishes that for a given $x^*$, a DC derived from a feasible but non-optimal follower solution does not have to be dominated by a DC derived from an optimal follower solution.

#### Theorem 2

Let $(x^*,y^*)\in \mathcal {P}$ be a bilevel-infeasible solution such that $\varOmega (x^*) \ne \emptyset$ and such that $F(x^*) \setminus \varOmega (x^*) \ne \emptyset$. Let ${{\hat{y}}}_1 \in \varOmega (x^*)$ and ${{\hat{y}}}_2 \in F(x^*) {\setminus } \varOmega (x^*)$. Then, there exist instances where the two DCs, one derived from $\mathcal {D}({\hat{y}}_1,\mathcal {P})$ and the other derived from $\mathcal {D}({\hat{y}}_2,\mathcal {P})$, do not dominate one another.

#### Proof

To prove this result, we consider an adaptation of the famous example from [43], namely

$$\begin{aligned} \min _{x\in {\mathbb {Z}}} \{ x-y : y \in \varOmega (x) \}, \end{aligned}$$

where $\varOmega (x)$ is the set of optimal solutions of the problem

9a $$\begin{aligned} \min _{y\in {\mathbb {Z}}} \{ y^2 :~ 25x -20y&\ge -30 \end{aligned}$$

9b $$\begin{aligned} -x -2 y&\ge -10 \end{aligned}$$

9c $$\begin{aligned} -2 x + y&\ge -4 \end{aligned}$$

9d $$\begin{aligned} 2 x + 10y&\ge 15 \ \}, \end{aligned}$$

and $\mathcal {P}$ is the set of feasible solutions to the linear constraints (9a)–(9d).

For $x^*=2$, we have ${{\hat{y}}}_1 = 2 \in \varOmega (x^*)$ and ${{\hat{y}}}_2=3 \in F(x^*) \setminus \varOmega (x^*)$. The disjunctions associated with ${{\hat{y}}}_1 = 2$ are

$$\begin{aligned}&\mathcal {D}_0({\hat{y}}_1): y^2 \le 4,{} & {} \mathcal {D}_1(\hat{y_1}): x \le 9/25,{} & {} \mathcal {D}_2(\hat{y_1}): x \ge 7, \\{} & {} {}&\mathcal {D}_3(\hat{y_1}): x \ge 7/2,{} & {} \mathcal {D}_4(\hat{y_1}): x \le -3. \end{aligned}$$

Note that both ${\mathcal {P}} \cap \mathcal {D}_2(\hat{y_1})$ and ${\mathcal {P}} \cap \mathcal {D}_4(\hat{y_1})$ are empty. When (CG-SOCP) is solved using the cut-coefficient normalization with 1-norm (see Sect. 3.5 for more details on normalization) for the solution $x^*=2$ and $y^*=4$, the DC obtained is $-1.25x+3.1y\le 5.7$.

Similarly, the disjunctions associated with ${{\hat{y}}}_2 = 3$ are

$$\begin{aligned}&\mathcal {D}_0(\hat{y_2}): y^2 \le 9,{} & {} \mathcal {D}_1(\hat{y_2}): x \le 29/25,{} & {} \mathcal {D}_2(\hat{y_2}): x \ge 5, \\{} & {} {}&\mathcal {D}_3(\hat{y_2}): x \ge 4,{} & {} \mathcal {D}_4(\hat{y_2}): x \le -8. \end{aligned}$$

**Fig. 1:**
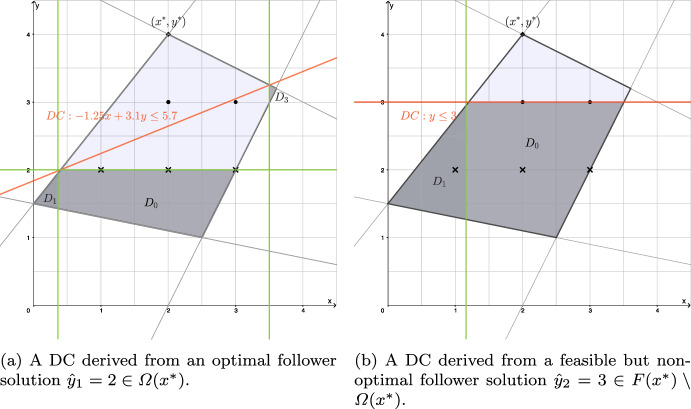
An example illustrating two DCs which do not dominate one another

Note that ${\mathcal {P}} \cap \mathcal {D}_i(\hat{y_2})$ is empty for all $i\in \{2,3,4\}$. The corresponding DC obtained by using cut-coefficient normalization with 1-norm and the solution $x^*=2$ and $y^*=4$ is $y\le 3$. Figure 1 illustrates the two cuts, neither of which dominates the other.

$$\square $$

Theorem 2 indicates that multiple DCs which do not dominate one another could be derived when separating a bilevel-infeasible point $(x^*,y^*)$. Moreover, it also means that we do not need to solve the follower problem to optimality in order to generate a (potentially) non-dominated DC. This is exploited in one of the separation procedures described in the next section.

### Separation procedures

We now turn our attention to describing how to computationally separate our DCs for a solution $(x^*,y^*) \in \mathcal {P}$. Note that we do not necessarily need the optimal solution of the follower problem (4) for $x=x^*$ to be able to cut off a bilevel-infeasible solution $(x^*,y^*)$, as any ${{\hat{y}}}$ that is feasible for the follower problem with $q({{\hat{y}}})< q(y^*)$ gives a violated DC as described in Theorem 1. Motivated by the result of Theorem 2, we implement two different strategies for separation which are described in Algorithm 1.

In the first one, denoted as O, we solve the follower problem to optimality, and use the optimal ${{\hat{y}}}$ in (CG-SOCP). In the second strategy, denoted as G, for each feasible integer follower solution ${{\hat{y}}}$ with a better objective value than $q(y^*)$ obtained during solving the follower problem, we try to solve (CG-SOCP). The procedure returns the first-found significantly-violated cut, i.e., it finds a DC greedily. A cut $\alpha 'x +\beta 'y \ge \tau$ is considered to be *significantly violated* by $(x^*,y^*)$ if $\tau - \alpha 'x^* -\beta 'y^*> \varepsilon$ for some $\varepsilon >0$.

If $(x^*,y^*)$ is a bilevel-infeasible solution satisfying integrality constraints, Algorithm 1 returns a violated cut with both strategies. Otherwise, i.e., if $(x^*,y^*)$ is not integer, a cut may not be obtained for the reasons discussed after Theorem 1.
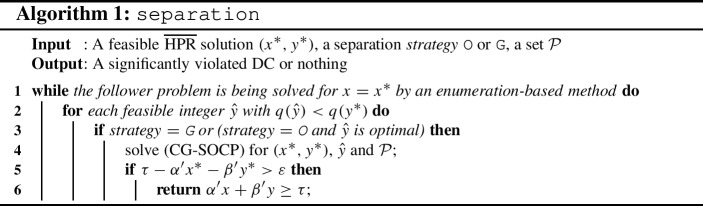


### Removing redundant disjunctions

The examples given in the proof of Theorem 2 illustrate that removing redundant disjunctions could lead to faster separation and also to DCs which dominate the DCs obtained without the removal of such disjunctions:

- For ${{\hat{y}}}_2$, the sets ${\mathcal {P}} \cap D_i({{\hat{y}}}_2)$ for $i \in \{2,3,4\}$ are empty. Thus we do not need to consider these disjunctions when defining (CG-SOCP), which leads to a smaller SOCP in the separation procedure.
- For ${{\hat{y}}}_1$, the set ${\mathcal {P}} \cap D_3({{\hat{y}}}_1)$ does not contain any bilevel-feasible solution, as it does not contain any integer solution. By removing the disjunction $D_3({{\hat{y}}}_1)$, a new DC $y \le 2$ can be obtained by using cut-coefficient normalization with 1-norm. This new DC dominates the DC obtained when $D_3({{\hat{y}}}_1)$ is included.

Thus, ideally, we would like to eliminate disjunctions $\mathcal {D}_i({{\hat{y}}})$ which do not contain any bilevel-feasible solution. Because this condition is very difficult to verify, as pointed out in [19, cf. Theorem 5 and Corollary 1], we could simply check whether $\mathcal {D}_i({{\hat{y}}})$ is not satisfied for any point satisfying the variable bounds of $\mathcal {P}$ (thus, relaxing the condition of checking whether $\mathcal {D}_i({{\hat{y}}})$ is not satisfied for any point in $\mathcal {P}$) for $i = 1, \dots , m_2$. In particular, we could check whether

$$\begin{aligned} \sum _{j=1}^{n_1} {{\tilde{x}}}_j > f_i - B^i {\hat{y}} \end{aligned}$$

holds, where ${{\tilde{x}}}_j:= \min \{ A^i_j x^+_j, A^i_j x^-_j\}$ and where $x^+_j$ and $x^-_j$ are the upper and lower bounds imposed on *x* inside of $\mathcal {P}$, respectively.

However, this only considers each disjunction with variable bounds individually and may not be very effective. In what follows, we propose several other approaches, ordered by their computational effort.

*Relaxation-based removal.* A disjunction $\mathcal {D}_i({{\hat{y}}})$ is redundant if $\mathcal {P} \cap \mathcal {D}_i({{\hat{y}}}) = \emptyset$. Checking this requires solving a (small) SOCP.  If the disjunction can be removed, (CG-SOCP) is smaller. Moreover, each DC which can be obtained when the disjunction is considered in (CG-SOCP), can also be obtained when the disjunction is removed before solving (CG-SOCP).

*Integrality-based removal.* A disjunction $\mathcal {D}_i({{\hat{y}}})$ is redundant if $\mathcal {P} \cap \mathcal {D}_i({{\hat{y}}}) \cap {\mathbb {Z}}^n = \emptyset$. Checking this requires solving a (small) integer-SOCP. If such a disjunction is removed, (CG-SOCP) is smaller and the (potentially) resulting DC can dominate the DC obtained without removal of the disjunction (see, e.g., the example discussed above for ${{\hat{y}}}_1$).

*Optimality-based removal.* A disjunction $\mathcal {D}_i({{\hat{y}}})$ is redundant if among the solutions in $\mathcal {P} \cap \mathcal {D}_i({{\hat{y}}}) \cap {\mathbb {Z}}^n$, there is no solution that improves the current best objective-function value (say, $U\!B$) of the leader.

#### Theorem 3

Let $U\!B$ be the objective-function value of the best-known feasible solution for the bilevel program (1). Let furthermore ${{\bar{U}}}_i$ be the optimal objective-function value of the problem

$$\begin{aligned} \min \{ c'x + d' y: (x,y) \in \mathcal {P} \cap \mathcal {D}_i ({{\hat{y}}}) \cap {\mathbb {Z}}^n \}. \end{aligned}$$

If ${{\bar{U}}}_i \ge U\!B$, then the disjunction $\mathcal {D}_i({{\hat{y}}})$ is redundant and can be discarded from $D({\hat{y}},\mathcal {P})$ and thus also from (CG-SOCP).

#### Proof

If ${{\bar{U}}}_i \ge U\!B$, then any solution in $\mathcal {P} \cap \mathcal {D}_i({{\hat{y}}}) \cap {\mathbb {Z}}^n$ is not better for the bilevel program (1) than the best-known feasible solution. Thus any better solution cannot be in $\mathcal {P} \cap \mathcal {D}_i({{\hat{y}}}) \cap {\mathbb {Z}}^n$ and therefore no optimal solution is missed by removing $\mathcal {D}_i({{\hat{y}}})$.

$$\square $$

**Fig. 2:**
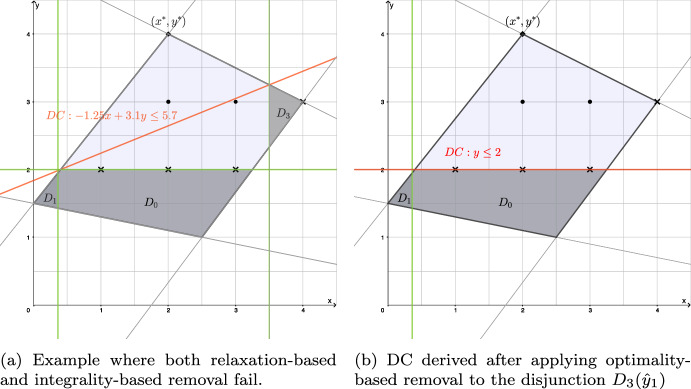
An example illustrating optimality-based removal of disjunctions

If the disjunction can be removed, (CG-SOCP) is smaller and the (potentially) resulting DC can dominate the DC obtained without removal of the disjunction. In particular, the removal can result in such a cut even if the integrality-based removal fails to remove a disjunction: For example, consider a slightly modified version of the instance in the proof of Theorem 2, where constraint (9c) is replaced by $-4x+3y\ge -7$. In this case, for ${{\hat{y}}}_1=2$, we have $\mathcal {D}_3(\hat{y_1}): x \ge 7/2$ and the set ${\mathcal {P}} \cap D_3({{\hat{y}}}_1)$ contains an integer solution, namely (4, 3). Thus, with the first two approaches of removing redundant disjunctions, we keep the disjunction $D_3({{\hat{y}}}_1)$ and would get the DC $-1.25x+3.1y\le 5.7$ using cut-coefficient normalization with 1-norm (see Fig. 2a). However, suppose that we know, e.g., of the bilevel-feasible solution (2, 2) which has the leader objective-function value $U\!B=0$. Because ${{\bar{U}}}_3=1$, the condition of Theorem 3 is satisfied, and the disjunction can be removed. The resulting DC in this case is $y\le 2$ using cut-coefficient normalization with 1-norm (see Fig. 2b).

### Normalization

As mentioned before, we need to deal with the fact that the feasible region of (CG-SOCP) is a cone. So (CG-SOCP) either has its optimum at the origin (implying that $(x^*,y^*)$ cannot be separated), or (CG-SOCP) is unbounded, implying that there is a violated inequality, which of course we could scale by any positive number so as to make the violation as large as we like. The standard remedy for this is to introduce a normalization constraint to (CG-SOCP). A typical good choice (see [20]) is to additionally impose

$$\begin{aligned} \Vert ((\bar{\pi _i})_{i=0}^{m_2}, \; (\tilde{\pi }_i)_{i=0}^{m_2}, \; \sigma , \; \rho )\Vert _1\ \le 1 \end{aligned}$$

on (CG-SOCP), i.e., that the 1-norm of the set of dual multipliers is unity. Because we are using a conic solver, we can alternatively impose

$$\begin{aligned} \Vert ((\bar{\pi _i})_{i=0}^{m_2}, \; (\tilde{\pi }_i)_{i=0}^{m_2}, \; \sigma , \; \rho )\Vert _2\ \le 1, \end{aligned}$$

which is just one constraint for a conic solver. This kind of normalization, where the norm of the vector of all dual variables is bounded by 1, is called the *standard* normalization (see, e.g., Lodi et al. [38]).

In [38], not only the standard normalization but also *uniform* normalization is concluded to be among the best normalizations in terms of numerical robustness. In uniform normalization, only the norm of the vector of dual variables corresponding to the constraints shared by all disjunctions (and not to the ones defining the disjunctions) is bounded, i.e.,

$$\begin{aligned} \Vert ((\bar{\pi _i})_{i=0}^{m_2}, \; (\tilde{\pi }_i)_{i=0}^{m_2} )\Vert _p\ \le 1, \end{aligned}$$

where $p \in \{1,2\}$. Note, that [38] considered only a generalization of the 1-norm, and not the 2-norm, for both the standard and the uniform normalization.

Another alternative is *cut-coefficient* normalization, where the norm of the cut-coefficients $\alpha$ and $\beta$ is bounded by one; so

$$\begin{aligned} \Vert (\alpha , \; \beta )\Vert _p\ \le 1 \end{aligned}$$

is imposed, typically for some $p \in \{1,2\}$. This may seem to be the most intuitive kind of normalization, as solving the (CG-SOCP) yields the desired cut-coefficients.

*Theoretical considerations concerning normalization.* To investigate the influence of normalization, we next present the duals of (CG-SOCP). Without normalization, the dual has objective function zero and the feasible region is

10a $$\begin{aligned}&\textstyle \sum _{i=0}^{m_2}x_i = x^* \end{aligned}$$

10b $$\begin{aligned}&\textstyle \sum _{i=0}^{m_2}y_i = y^* \end{aligned}$$

10c $$\begin{aligned}&\textstyle \sum _{i=0}^{m_2}\lambda _i = 1 \end{aligned}$$

10d $$\begin{aligned}&\lambda _i \ge 0{} & {} \forall i =0,\ldots , m_2 \end{aligned}$$

10e $$\begin{aligned}&{\bar{M}}x_i + {\bar{N}}y_i \ge \lambda _i{\bar{h}}{} & {} \forall i =0,\ldots , m_2 \end{aligned}$$

10f $$\begin{aligned}&{\tilde{M}}x_i + {\tilde{N}}y_i - \lambda _i {\tilde{h}} \in \mathcal {K^{**}}{} & {} \forall i =0,\ldots , m_2 \end{aligned}$$

10g $$\begin{aligned}&{\tilde{D}}y_0 - \lambda _0{\tilde{c}} \in \mathcal {Q}\end{aligned}$$

10h $$\begin{aligned}&A^i x_i \le \lambda _i (f_i - B^i {\hat{y}} - 1){} & {} \forall i =1,\ldots , m_2, \end{aligned}$$

i.e., the dual tries to find points $(x_i,y_i)$, such that $(x^*,y^*)$ is the sum of these points, and such that either $\lambda _i = 0$ or $\lambda _i > 0$ and $(\frac{1}{\lambda _i}x_i,\frac{1}{\lambda _i}y_i) \in \mathcal {P} \cap \mathcal {D}_i({\hat{y}})$. As a consequence, (10) is feasible if and only if $(x^*,y^*)$ is in ${{\,\textrm{conv}\,}}\left( \bigcup _{i=0}^{m_2} (\mathcal {P} \cap \mathcal {D}_i({\hat{y}})) \right)$. Note that this corresponds exactly to the case that the primal (CG-SOCP) does not find a violated cut, i.e., its optimal objective-function value is zero.

When deriving the duals of (CG-SOCP) with normalization, we assume that the normalization was imposed with the *p*-norm for $p \in \{1,2\}$. Let the $p^*$-norm be the dual norm of the *p*-norm, i.e. $p^* = 2$ for $p=2$ and $p^* = \infty$ for $p=1$. Note that normalizing (CG-SOCP) using the *p*-norm leads to a $p^*$-norm in the objective function of the dual.

In case of standard normalization, the dual of (CG-SOCP) is

11a $$\begin{aligned} -&\min \Vert (({\bar{\mu }}_i)_{i=0}^{m_2}, \; (\tilde{\mu }_i)_{i=0}^{m_2}, \; \mu _\sigma , \; \mu _\rho )\Vert _{p^*}\end{aligned}$$

11b $$\begin{aligned} {{\,\mathrm{s.t.}\,}}~&(10a)-(10d) \end{aligned}$$

11c $$\begin{aligned}&{\bar{M}}x_i + {\bar{N}}y_i \ge \lambda _i{\bar{h}} + {\bar{\mu }}_i{} & {} \forall i =0,\ldots , m_2\end{aligned}$$

11d $$\begin{aligned}&{\tilde{M}}x_i + {\tilde{N}}y_i - \lambda _i {\tilde{h}} - \tilde{\mu }_i \in \mathcal {K^{**}}{} & {} \forall i =0,\ldots , m_2 \end{aligned}$$

11e $$\begin{aligned}&{\tilde{D}}y_0 - \lambda _0{\tilde{c}} - \mu _\rho \in \mathcal {Q}\end{aligned}$$

11f $$\begin{aligned}&A^i x_i \le \lambda _i (f_i - B^i {\hat{y}} - 1) - {\mu _\sigma }_i{} & {} \forall i =1,\ldots , m_2. \end{aligned}$$

#### Observation 1

The problem (11) is always feasible, and there is a feasible interior point due to the free variables $({\bar{\mu }}_i)_{i=0}^{m_2}$, $(\tilde{\mu }_i)_{i=0}^{m_2}$, $\mu _\sigma$ and $\mu _\rho$, which relax the constraints to be in $\mathcal {P}$ and to be in $\mathcal {D}_i({\hat{y}})$. The optimal objective-function value of (11) is zero if and only if $(x^*,y^*)$ is in ${{\,\textrm{conv}\,}}\left( \bigcup _{i=0}^{m_2} (\mathcal {P} \cap \mathcal {D}_i({\hat{y}})) \right)$, i.e., if and only if there is no violated DC for $(x^*,y^*)$.

Note that Observation 1 is compatible with what is observed in [38] when deriving split-cuts for mixed-integer SOCP using disjunctive programming with SOCP.

For the uniform normalization, the dual of (CG-SOCP) is 12a12b

#### Observation 2

The problem (12) is not necessarily feasible, because only the constraints to be in $\mathcal {P}$ are relaxed with the variables $({\bar{\mu }}_i)_{i=0}^{m_2}$ and $(\tilde{\mu }_i)_{i=0}^{m_2}$. To be more precise, (12) is feasible if and only if $(x^*,y^*)$ is in ${{\,\textrm{conv}\,}}\left( \bigcup _{i=0}^{m_2} \mathcal {D}_i({\hat{y}}) \right)$. Due to the structure of our disjunctions (i.e., they are based on the follower constraints and the follower objective function), the point $(x^*,y^*)$ may not be in ${{\,\textrm{conv}\,}}\left( \bigcup _{i=0}^{m_2} \mathcal {D}_i({\hat{y}}) \right)$ and thus (12) could be infeasible.

Furthermore, as for standard normalization, the optimal objective-function value of (12) is zero if and only if $(x^*,y^*)$ is in ${{\,\textrm{conv}\,}}\left( \bigcup _{i=0}^{m_2} (\mathcal {P} \cap \mathcal {D}_i({\hat{y}})) \right)$.

We note that Observation 2 is different compared to what the authors of [38] obtain in their setting, as the convex hull of the disjunction for split cuts is $\mathbb R^n$ and thus their resulting problem (12) is always feasible.

For the cut-coefficient normalization, the dual of (CG-SOCP) is 13a13b13c13d so geometrically (13) determines a point in ${{\,\textrm{conv}\,}}\left( \bigcup _{i=0}^{m_2} (\mathcal {P} \cap \mathcal {D}_i({\hat{y}})) \right)$ that minimizes the distance (in $p^*$-norm) to $(x^*,y^*)$.

#### Observation 3

Problem (13) is feasible if and only if ${{\,\textrm{conv}\,}}\left( \bigcup _{i=0}^{m_2} (\mathcal {P} \cap \mathcal {D}_i({\hat{y}})) \right)$ is non-empty. If (13) is infeasible, then all disjunctions are empty (i.e., redundant as described in Sect. 3.4).

As a result of the investigation of the normalization, we know that Assumption 3 is always satisfied with standard normalization. Unfortunately this is not the case with uniform and cut-coefficient normalization. We describe in Sect. 5.3 how we deal with this.

## Solution methods using disjunctive cuts

We now present two solution methods based on DCs: one applicable for the general bilevel program (1), and one dedicated to a binary version of (1).

### A branch-and-cut algorithm

We propose to use the DCs in a B &C algorithm to solve the bilevel program (1). The B &C can be obtained by modifying any given continuous-relaxation-based B &B algorithm to solve the HPR (assuming that there is an off-the-shelf solver for $\overline{\text{ HPR }}$ that always returns an extreme optimal solution $(x^*,y^*)$ like e.g., a simplex-based B &B for a linear $\overline{\text{ HPR }}$^1^).

In particular, we adapt the B &B algorithm in the following way: Use $\overline{\text{ HPR }}$ as initial relaxation ${\mathcal {P}}$ at the root-node of the B &C. Whenever a solution $(x^*,y^*)$ which is integer is encountered in a B &C node, call the DC separation. If a violated DC is found, add the DC to the set ${\mathcal {P}}$ (which also contains, e.g., variable fixing by previous branching decisions, previously added globally or locally valid DCs, ...) of the current B &C node, otherwise the solution is feasible and the incumbent can be updated. Note that DCs are only locally valid except the ones from the root node, because $\mathcal {P}$ includes branching decisions. If $\mathcal {P}$ is empty or optimizing over $\mathcal {P}$ leads to an objective-function value that is greater than the objective-function value of the current incumbent, we fathom the current node. In our implementation, we also use DC separation for fractional $(x^*,y^*)$ as described in Sect. 3.2 for strengthening the relaxation.

#### Theorem 4

The B &C solves the bilevel program (1) in a finite number of B &C-iterations under our assumptions.

#### Proof

First, suppose that the B &C terminates, but the integer solution $(x^*,y^*)$ is not bilevel feasible. This is not possible, as by Theorem 1 and the observations thereafter, the DC generation procedure finds a violated cut to cut off the integer point $(x^*,y^*)$ in this case.

Next, suppose that the B &C terminates and the solution $(x^*,y^*)$ is bilevel feasible, but not optimal. This is not possible, because by construction, the DCs never cut off any bilevel-feasible solution (or in case of optimality-based removal, any bilevel-feasible solution, which has a better leader objective-function value than the currently best-known solution) of the current subtree.

Finally, suppose that the B &C never terminates. This is not possible, as all variables are integer and bounded due to Assumption 1, thus there is only a finite number of nodes in the B &C tree. Moreover, this means that there is also a finite number of integer points $(x^*,y^*)$, thus we solve the follower problem and (CG-SOCP) a finite number of times. The follower problem is discrete and can therefore be solved in a finite number of iterations.

$$\square $$

### A cutting-plane algorithm for binary IBNPs

The DCs can be directly used in a cutting-plane algorithm under the following assumption.

#### Assumption 4

All variables in the bilevel program (1) are binary variables.

The algorithm is detailed in Algorithm 2. It starts with the HPR as initial relaxation of VFR, which is solved to optimality. Then the chosen DC separation routine (either O or G) is called to check if the obtained integer-optimal solution is feasible for constraint (3e). If not, the obtained DC is added to the relaxation to cut off the optimal solution, and the procedure is repeated with the updated relaxation.

Due to Assumption 4, each obtained binary optimal solution is an extreme point of the convex hull of $\overline{\text{ HPR }}$, and thus due to Theorem 1, a violated cut will be produced by the DC separation if the solution is not bilevel feasible. Note that without Assumption 4, i.e., if variables are allowed to be integer and not just binary, an optimal solution may not be an extreme point of $\overline{\text{ HPR }}$. In this case, Theorem 1 does not apply. Thus, we cannot guarantee that the DC separation finds a violated cut. As a consequence, our proposed cutting-plane algorithm only works for binary instances.
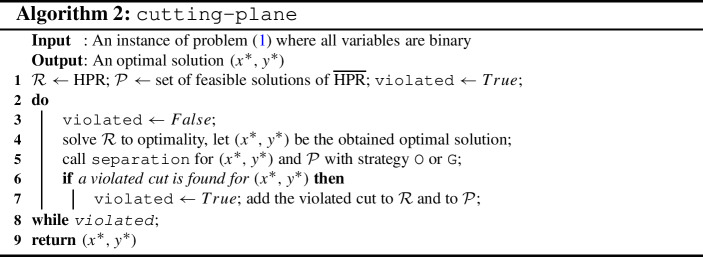


## Computational analysis

In this section, we present computational results to empirically compare methods and strategies proposed in Sects. 3 and 4. We also assess computational difficulties in the presence of multiple linking constraints, or in the presence of integer instead of binary variables. Finally, we compare our new DC-based branch-and-cut with the state-of-the-art MIBLP-solver MIX++ [18, 19].

### Instances

In our computations, we consider two sets of instances: the quadratic bilevel covering problem (QBCov) instances (originally studied in [22] and extended here with multiple linking constraints) and a new additional set of quadratic bilevel multiple knapsack problem (QBMKP) instances derived from the SAC-94 library [26]. The instances are available at https://msinnl.github.io/pages/instancescodes.html. All instances can be described as

14a $$\begin{aligned}&\min ~ \hat{c}' x + \hat{d}'y \end{aligned}$$

14b $$\begin{aligned} {{\,\mathrm{s.t.}\,}}&~\hat{M} x + \hat{N} y \ge \hat{h} \end{aligned}$$

14c $$\begin{aligned}&y \in \arg \min \{ y' \hat{R} y : \hat{A} x + \hat{B} y \ge \hat{f}, ~y \in \{0,1\}^{n_2} \}\end{aligned}$$

14d $$\begin{aligned}&x \in \{0,1\}^{n_1}, \end{aligned}$$

where $$\hat{c} \in {\mathbb {R}}^{ n_1}$$, $$\hat{d} \in {\mathbb {R}}^{ n_2}$$, $$\hat{M} \in {\mathbb {R}}^{m_1 \times n_1}$$, $$\hat{N} \in {\mathbb {R}}^{m_1 \times n_2}$$, $$\hat{h} \in {\mathbb {R}}^{ m_1}$$, $$\hat{R} =\hat{V}'\hat{V} \in {\mathbb {Z}}^{n_2 \times n_2}$$, $$\hat{A} \in {\mathbb {Z}}^{ m_2 \times n_1}$$, $$\hat{B} \in {\mathbb {Z}}^{m_2 \times n_2}$$, and $$\hat{f} \in {\mathbb {Z}}^{m_2}$$.

*The QBCov instances.* In this setting, we chose $m_2=1$, in which case the problem can be seen as the covering-version of the quadratic bilevel knapsack problem studied by Zenarosa et al. in [54]. Indeed, [54] considers a single leader variable ($n_1=1$) and no coupling constraints at the leader ($m_1=0$), and with a quadratic non-convex leader objective function. The linear variant of such a bilevel knapsack-problem is studied in, e.g., [9, 10]. We note that [9, 10, 54] propose problem-specific solution approaches.

We generated 40 random instances in the following way. We considered $n_1 = n_2$ for $n_1+n_2 = n\in \{20, 30, 40, 50\}$, and we study instances with no (as in [54]) and with one leader constraint (14b), so $m_1 \in \{0,1\}$. For each *n*, we created five random instances for each $m_1 \in \{0,1\}$. Furthermore, we chose all entries of $\hat{c}$, $\hat{d}$, $\hat{M}$, $\hat{N}$, $\hat{A}$, and $\hat{B}$ uniformly at random from $\{0,1,\dots ,99\}$. The values of $\hat{h}$ and $\hat{f}$ (which are scalars for these instances) were set to the sum of the entries of the corresponding rows in the constraint matrices divided by four. The matrix $\hat{V}\in {\mathbb {R}}^{n_2 \times n_2}$ has integer entries chosen uniformly at random from the set $\{0,1,\dots ,9\}$. We extended this data set from [22] with 40 new instances generated in the same way, by choosing $m_2=2$.

*The QBMKP instances.* These instances were derived from the multiple knapsack problem (MKP) instances from SAC-94 library [26] which is a benchmark library containing 0/1 MKP instances. From there we chose 50 instances and generated 300 new instances of the QBMKP as follows.

The instances have 2 to 10 constraints and 10 to 105 items. For each instance of this data set, we first constructed two different QBMKP instances by keeping all but the last $m_2$ constraints at the leader problem, where $m_2 \in \{1,2\}$. The first 50 or 75% of items are associated to leader variables *x*, the remaining ones are associated to follower variables *y*. The coefficients of the original MKP objective function are assigned to the leader. Each budget constraint of the starting MKP, say $a'x + b'y \le f$, is translated into a covering constraint of type (14b) as $a'x + b'y \ge e'a + e'b - \hat{f}$ (where *e* is the vector of all ones). To generate the positive semidefinite matrix ${{\hat{R}}}$ determining the follower objective function, we follow a procedure proposed in [30]. We first randomly generated quadratic matrices *V* of suitable size whose entries are chosen uniformly at random from $\{-\sigma , \dots , \sigma \}$ where $\sigma := \lceil \root 4 \of {||\hat{d}||_{\infty }} \rceil$ and then set ${{\hat{R}}}:= V'V$. This allows to keep the order of magnitude for the coefficients of the objective function of the follower similar to those of the leader. Following this procedure, we obtained 200 binary instances, 100 with one linking constraint and 100 with two linking constraints.

Lastly, we generated integer instances where $m_2=1$, all decision variables take value in $\{0,\ldots , 5\}$, and the right-hand side of the previously generated covering constraints are multiplied by two. Together with the two choices of variable assignments to the leader and the follower problems, we obtained 100 integer QBMKP instances.

*Linearization of instances.* The structure of (14) allows for an easy linearization of the convex nonlinear terms in the binary instances using a standard McCormick linearization to transform the starting problem into an MIBLP. This allows us to compare the performance of our algorithm against a state-of-the-art MIBLP-solver MIX++ from Fischetti et al. [18, 19].

### Computational environment

All experiments were executed on a single thread of an Intel Xeon E5-2670v2 machine with 2.5 GHz processor with a memory limit of 10 GB and a time limit of 600 s. Our B &C algorithm and our cutting-plane algorithm both are implemented in C++. They make use of IBM ILOG CPLEX 12.10 (in its default settings, except for disabling presolve so that we can access the original HPR formulation, setting the MIP gap tolerance to zero, and running it single-threaded) as branch-and-cut framework in our B &C algorithm and as solver for ${\mathcal {R}}$ in our cutting-plane algorithm. During the B &C, CPLEX’s internal heuristics are allowed and a bilevel-infeasible heuristic solution is just discarded if a violated cut cannot be obtained. For calculating the follower solution ${{\hat{y}}}$ for a given $x^*$, we also use CPLEX. For solving (CG-SOCP), we use MOSEK [44] 9.2 in its default settings, except for running it always single-threaded and using always the primal solver to avoid numerical issues. The solver MIX++ against which we compare was run with CPLEX 12.9, which is the newest CPLEX version compatible with this solver.

### Implementation details

*Update of* $\mathcal {P}$. For both the B &C and the cutting-plane algorithm, we start with $\overline{\text{ HPR }}$ as initial $\mathcal {P}$ and do not update it with dynamically added DCs. This is in line with the recent implementation of DCs with split-cuts for mixed-integer SOCP in [38], and prevents potential numerical instabilities. However, in the B &C we update $\mathcal {P}$ with the local variable bounds at the current node in the B &C-tree. Thus the obtained DCs are only locally valid (i.e., in the current subtree) and are added as *locally valid cuts*. Doing this is numerically safe, as variable bound constraints are already present in the original problem and they just need to be updated, i.e., the number of constraints remains the same.

We note that technically, in Theorem 1, we need that the current $\mathcal {P}$ incorporates all added DCs and the local variable bounds to ensure that $(x^*,y^*)$ is an extreme point, and thus can be separated in case it is bilevel infeasible. However, in our computational experiments, we never encountered any issues when not including previous DCs in $\mathcal {P}$. On the other hand, updating ${\mathcal {P}}$ with the local bounds was crucial to make the separation work for integer instances.

*Solving the follower problem to obtain* ${\hat{y}}$. During the separation of both integer and fractional points $(x^*,y^*)$, while solving the follower problem, we make use of the follower objective-function value $q(y^*)$, by setting it as an upper cutoff value. This is a valid approach because a violated DC exists only if $\Phi (x^*)<q(y^*)$.

Furthermore, we use the solution-pool feature of CPLEX. This means that CPLEX keeps feasible solutions obtained in previous iterations and tries to use them as initial solution. As a consequence, when using the separation option G, CPLEX may not need to start the solution process, as a solution from the solution-pool might be feasible for the current follower problem.

*Checking for redundant disjunctions.* In our implementation, we only check the redundancy of linear disjunctions, i.e., $\mathcal {D}_i({{\hat{y}}})$ for $i=1,\ldots , m_2$, because we observed in the preliminary tests that the objective disjunction is almost never redundant as $(x^*,{\hat{y}})$ usually gives a feasible solution to $\mathcal {P} \cap \mathcal {D}_i({{\hat{y}}})$.

When solving the problems to detect redundancy, we keep the original leader objective function and define the feasible region as $\mathcal {P} \cap \mathcal {D}_i({{\hat{y}}})$ or $\mathcal {P} \cap \mathcal {D}_i({{\hat{y}}})\cap {\mathbb {Z}}^n$ together with the local variable bounds which will be used to obtain a DC. For optimality-based removal, we solve the problem with an upper cutoff value $z^*-10^{-5}$, where $z^*$ is the current leader incumbent objective value. Note that redundancy of a disjunction is indicated by infeasibility of the corresponding detection problem. Thus as soon as CPLEX finds a feasible solution for a detection problem, we know that the disjunction is not redundant. Hence, we set the CPLEX parameter solution limit to one.

*Solving (*CG-SOCP*).* To avoid numerical issues, whenever a coefficient of a DC is close enough to zero (i.e., absolute value less than $5\cdot 10^{-6}$), we round it to zero and adapt the right-hand side of the DC to maintain a valid cut.

Unless mentioned differently, we use standard normalization with the 2-norm, where Assumption 3 is satisfied. Whenever the dual of (CG-SOCP) is infeasible in the case of uniform normalization, we take $\alpha$, $\beta$ and $\tau$ from an unbounded ray of the primal (which is provided by MOSEK) as DC. To prevent numerical issues, we scale any unbounded ray in such a way that $\Vert (\alpha , \beta )\Vert _2 = 1$ holds. If the dual of (CG-SOCP) with cut-coefficient normalization is infeasible, all disjunctions are empty as described in Sect. 3.5. Thus we add the always violated cut $\alpha = 0$, $\beta =0$ and $\tau = 1$ in this case.

*Separation.* We set the minimum acceptable cut violation $\varepsilon$ described in Sect. 3.3 to $10^{-6}$ for our experiments. We control the number of cuts added for separating fractional points as follows. At the root node of the B &C tree, we add as many cuts as needed, i.e., we check if we are able to cut off the current point with a DC until no violated cut can be obtained. At all other nodes, we add at most one DC and then proceed to branching, as the separation procedure could be time consuming.

Finally, in our B &C implementation, we also have to deal with integer solutions that are produced by the internal heuristics of CPLEX. In this case, we do not necessarily have a useful ${\mathcal {P}}$ for separation at hand. Thus, if the produced heuristic solution is bilevel infeasible and we fail to cut it off with a DC, we just use the reject-feature of CPLEX to reject this solution (this prevents CPLEX from updating the incumbent with the heuristic solution).

### Numerical results

We start by assessing the performance of the B &C approach, and by evaluating how the choice of separation strategy, removal of redundant disjunctions, and normalization affect the overall performance. We then compare the B &C against two alternatives: the cutting-plane method described in Sect. 4 and the state-of-the-art MIBLP solver MIX++ from [18, 19]. We conducted these experiments on 140 instances from our benchmark set that contain only binary variables, and a single linking constraint. Finally, we extended the benchmark set, and we also demonstrate the performance of our B &C when applied to instances with multiple linking constraints, and with integer variables.

*Performance of different ingredients of the B &C algorithm.* We discussed different separation procedures in Sect. 3.2. While executing our B &C algorithm, we consider four different settings for the separation of cuts:

- IO: only integer solutions are separated using strategy O,
- IFO: both integer and fractional solutions are separated using strategy O,
- IG: only integer solutions are separated using strategy G,
- IFG: both integer and fractional solutions are separated using strategy G.

**Fig. 3:**
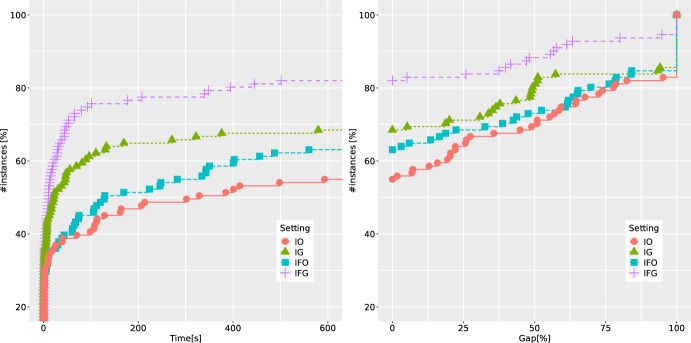
ECDFs reporting runtimes and final gaps for four different separation strategies of the B &C, over binary instances with one linking constraint

**Fig. 4:**
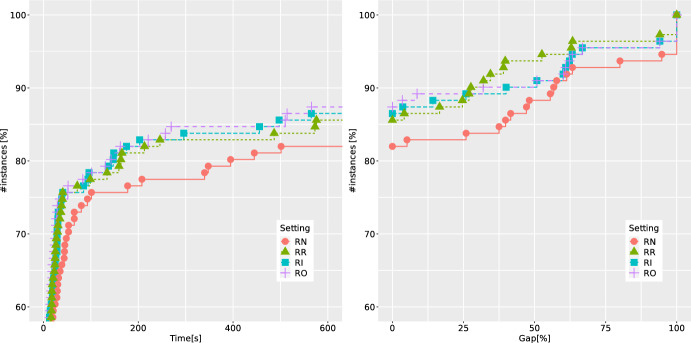
ECDFs reporting runtimes and final gaps for four strategies for the removal of redundant disjunctions, over binary instances with one linking constraint

**Fig. 5:**
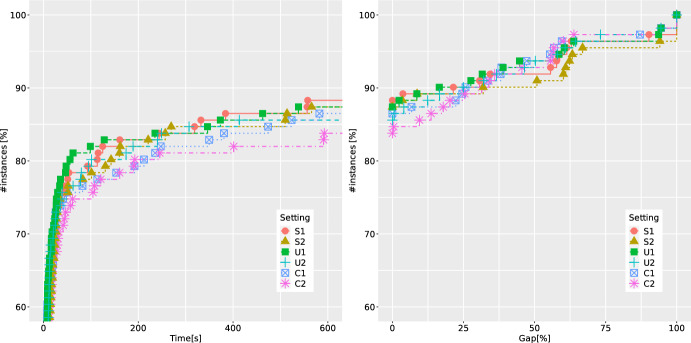
ECDFs reporting runtimes and final gaps for six different normalization strategies for (CG-SOCP), over binary instances with one linking constraint

**Fig. 6:**
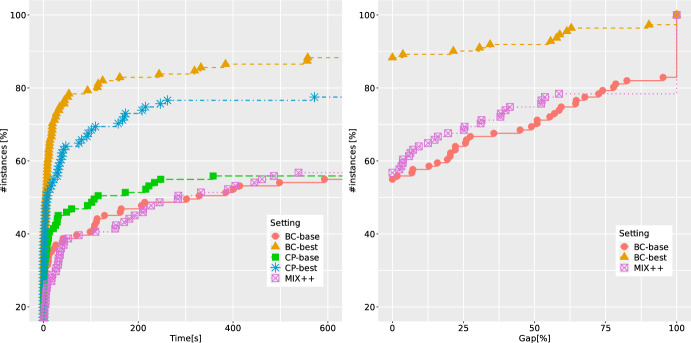
ECDFs reporting runtimes and final gaps for the base and the best versions of the B &C and cutting-plane methods, and the MIBLP solver, over binary instances with one linking constraint

In Fig. 3 we compare these four settings for the B &C. We show the empirical cumulative distribution functions (ECDFs) w.r.t. the runtimes and final gaps. The gaps are defined in the following way. If an instance is proven to be infeasible, we define the gap to be zero. Otherwise, if no feasible solution is found, we define the gap to be 100. If a feasible solution is found, then the gap is calculated as $100 (z^*-LB)/z^*$, where $z^*$ and *LB* are the best-known objective-function value of a feasible solution and the lower bound, respectively. Note that by the construction of our instances, the value zero is a trivial lower bound for all feasible instances. Thus the gaps will always range between zero and 100. The ECDFs with e.g., runtimes can be interpreted as the percentage of instances (shown in *y*-axis) that can be solved within a certain amount of time (depicted in the *x*-axis). In order to have a fair comparison, out of 140 instances from this benchmark set, we only consider 111 instances for which at least one of the methods was either able to find a feasible solution, or to prove infeasibility (for 29 instances from QBMKP, the feasibility status remains unknown). We observe that the best-performing setting is IFG. This can be explained by the fact that non-optimal follower solution may also provide a strong DCs (cf. Theorem 2) and by the significant savings in separation time (as we are avoiding to solve the follower problem to optimality). Our preliminary results reported in [22] did not identify IFG as the best setting, because there was no control mechanism implemented to limit the number of separated DCs for each fractional point, which may cause overloading of the master problem. This is now regulated as described in Sect. 5.3.

**Table 1:** Average results of five methods over 40 QBCov instances with one linking constraint

*n*	Setting	*t*	Gap	Gap*	RGap	RGap*	nNode	nICut	nFCut	nRed	$t_F$	$t_S$	nSol
20	BC-base	2.0	0.0	0.0	56.0	16.2	165.8	61.9	–	–	1.3	0.4	10/10
	BC-best	1.1	0.0	0.0	66.6	16.4	93.0	17.1	46.1	40.3	0.4	0.3	10/10
	CP-base	1.0	–	0.0	–	–	–	15.3	–	–	0.3	0.2	10/10
	CP-best	0.2	–	0.0	–	–	–	10.6	–	1.0	0.0	0.1	10/10
	MIX++	2.2	0.0	0.0	14.5	14.5	25.0	–	–	–	–	–	10/10
30	BC-base	154.8	2.6	0.9	70.5	15.1	4461.1	1879.9	–	–	143.8	10.0	9/10
	BC-best	3.6	0.0	0.0	53.4	14.0	507.8	32.8	285.9	246.8	1.5	1.7	10/10
	CP-base	70.7	–	0.0	–	–	–	131.3	–	–	12.5	0.8	10/10
	CP-best	16.7	–	0.0	–	–	–	77.4	–	1.1	0.3	0.4	10/10
	MIX++	116.5	0.0	0.0	15.6	15.6	300.2	–	–	–	–	–	10/10
40	BC-base	305.4	16.3	7.7	100.0	21.1	5717.0	2058.4	–	–	289.4	14.7	6/10
	BC-best	25.0	0.0	0.0	90.0	18.9	3282.1	196.2	1759.9	1629.5	11.0	12.7	10/10
	CP-base	256.2	–	2.3	–	–	–	249.2	–	–	52.6	1.9	6/10
	CP-best	117.6	–	0.9	–	–	–	182.4	–	0.7	0.9	1.2	9/10
	MIX++	456.9	20.2	8.7	32.2	21.8	368.2	–	–	–	–	–	4/10
50	BC-base	551.2	46.9	27.8	100.0	36.2	7513.8	2618.2	–	–	526.4	23.2	2/10
	BC-best	233.6	3.1	2.8	100.0	33.5	23479.3	1073.1	14859.2	9488	95.5	128.1	9/10
	CP-base	549.3	–	17.9	–	–	–	418.8	–	–	153.8	3.9	1/10
	CP-best	423.1	–	11.8	–	–	–	374.7	–	0.5	2.0	3.1	4/10
	MIX++	600.0	53.1	31.6	57.8	38.1	245.5	–	–	–	–	–	0/10

**Table 2:** Average results of five methods over 71 QBMKP instances with one linking constraint

$n_1/n$	Setting	*t*	Gap	Gap*	RGap	RGap*	nNode	nICut	nFCut	nRed	$t_F$	$t_S$	nSol
0.50	BC-base	417.5	34.2	23.8	85.8	30.0	5847.7	2497.0	–	–	403.4	13.0	11/26
	BC-best	71.2	3.6	3.6	78.6	13.6	5151.6	342.3	6415.3	4874.7	31.0	36.4	24/26
	CP-base	357.2	–	20.7	–	–	–	257.4	–	–	131.2	5.3	12/26
	CP-best	155.3	–	19.2	–	–	–	164.5	–	39.8	0.8	0.9	19/26
	MIX++	341.2	20.4	14.3	35.9	22.4	199.5	–	–	–	–	–	16/26
0.75	BC-base	302.8	38.2	18.1	66.3	23.7	17841.1	6581.2	–	–	258.9	39.6	23/45
	BC-best	155.1	14.5	7.0	62.8	17.8	9657.0	600.8	9427.2	5600.4	63.2	84.1	35/45
	CP-base	294.2	–	14.8	–	–	–	171.0	–	–	66.4	1.4	23/45
	CP-best	191.5	–	10.7	–	–	–	154.2	–	8.7	0.8	1.3	31/45
	MIX++	316.0	38.4	14.6	45.7	20.6	712.7	–	–	–	–	–	23/45

In what follows, we continue with the setting IFG, and investigate how the potential removal of redundant disjunctions (discussed in Sect. 3.4) affects the overall performance. Two additional ECDFs are reported in Fig. 4 for the settings denoted as RN (no removal of redundant disjunctions), RR (relaxation-based removal), RI (integrality-based removal), and RO (optimality-based removal). We notice that the runtime can be improved, even when only the simplest strategy RR is applied. Also, the other more computationally-expensive removal strategies do not deteriorate the runtime, and in particular, they help to significantly improve the final gaps. For example, when including the strongest strategy RO, for 90% of the instances, the final gap remains below 10%, whereas without the removal, the respective gap can be as large as 50%. Even though for the few most difficult instances, the best final gaps are obtained when using the RR strategy, we decided to continue with the rest of experiments using RO as a more stable and robust setting.

**Fig. 7:**
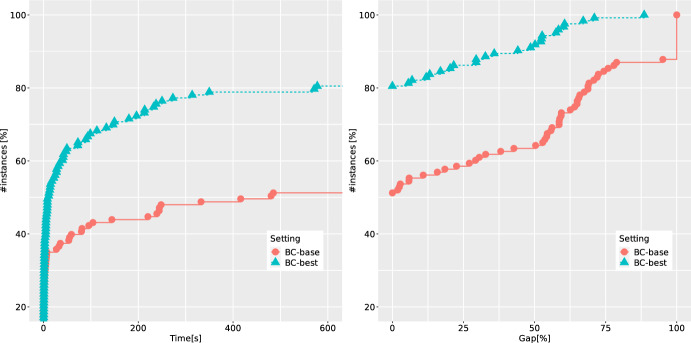
ECDFs reporting runtimes and final gaps for BC-base and BC-best over 123 binary instances with two linking constraints

Finally, when focusing on possible enhancement of the B &C procedure, we look at the effectiveness of normalization strategies for (CG-SOCP) presented in Sect. 3.5. The corresponding ECDFs are shown in Fig. 5. The letters S, U and C stand for standard, uniform and cut-coefficient normalization strategies, respectively, followed by $p \in \{1,2\}$ denoting the type of norm used. We observe that all normalization strategies perform very similarly, with two consistent trends: the worst-performing one is C (which is also in line with the known results from the literature on DCs, see, e.g., [38]), and the 2-norm is always outperformed by the 1-norm. The latter can be explained by the sparsity and better numerical stability of cuts produced using the 1-norm. Thus, based on these experiments, we decide to use the strategy IFG combined with RO and S1 as our best setting (denoted as BC-best in the following). We denote by BC-base the setting where IO is combined with RN and S2 (the setting which was also used in our earlier study in [22]).

**Table 3:** Average results of BC-base and BC-best over 40 QBCov instances with two linking constraints

*n*	Setting	*t*	Gap	Gap*	RGap	RGap*	nNode	nICut	nFCut	nRed	$t_F$	$t_S$	nSol
20	BC-base	2.0	0.0	0.0	90.0	26.3	291.1	108.9	–	–	1.2	0.7	10/10
	BC-best	2.0	0.0	0.0	90.0	25.7	174.6	26.1	90.6	139.4	0.8	0.7	10/10
30	BC-base	225.0	14.8	10.5	90.0	27.4	3919.3	1643.0	–	–	211.0	13.3	7/10
	BC-best	42.5	0.0	0.0	73.6	24.0	5280.2	216.1	3425.3	5379.6	19.8	20.7	10/10
40	BC-base	339.3	32.0	12.7	73.3	21.9	4469.1	1530.4	–	–	321.7	16.8	5/10
	BC-best	102.0	0.0	0.0	80.0	21.3	8361.7	575.6	5255.2	6928.8	44.6	54.0	10/10
50	BC-base	550.4	56.0	36.9	100.0	40.3	3299.2	1218.1	–	–	532.7	17.0	1/10
	BC-best	364.0	26.1	25.3	100.0	39.4	21184.3	1114.2	15539.7	17114.4	147.3	206.4	5/10

**Table 4:** Average results of BC-base and BC-best over 83 QBMKP instances with two linking constraints

$n_1/n$	Setting	*t*	Gap	Gap*	RGap	RGap*	nNode	nICut	nFCut	nRed	$t_F$	$t_S$	nSol
0.50	BC-base	417.8	40.1	19.8	86.0	26.0	3776.9	1387.8	–	–	404.8	12.3	14/33
	BC-best	191.4	8.2	8.2	79.3	16.9	10128.7	524.2	9623.2	12297.6	82.1	102.8	26/33
0.75	BC-base	295.6	27.8	14.4	67.9	17.7	15587.6	3987.9	–	–	246.7	46.2	26/50
	BC-best	163.0	9.1	8.9	58.4	15.4	9742.1	385.6	8017.7	10365.4	71.2	86.4	38/50

*Comparison against alternative methods.* For binary IBNPs we proposed an alternative cutting-plane algorithm in Sect. 4.2. This algorithm can be implemented with both separation strategies (G and O). Moreover, the removal of redundant disjunctions and normalization strategies can be fine-tuned as well. In Fig. 6, we compare the B &C results with the two settings (BC-best versus BC-base) against the results obtained by the cutting-plane algorithm (CP-best which involves G, RO, and S1 versus CP-base which involves O, RN, and S2) as well as a state-of-the-art MIBLP solver MIX++ of Fischetti et al. [18, 19], which is able to solve the linearized version of our instances. Figure 6 shows the ECDFs of the runtime and the final gaps at the end of the time limit. It can be seen that the best settings significantly improve their base counterparts. This is particularly pronounced for the B &C algorithm, where the base setting solves only 55% of instances to optimality, whereas its best counterpart increases this number to almost 90%. Similar (but not so drastic) improvements are obtained for the cutting-plane method too. Finally, the overall best-performing approach is BC-best and the solver MIX++ is also outperformed by both the cutting-plane algorithm and the B &C.

Tables 1 and 2 provide additional insights into this comparison. For each of the five methods, we report the following average values: the runtime in seconds (*t*), the final gap (Gap), the final gap with respect to the best-known upper bound (Gap*), the root gap (RGap), the root gap with respect to the best-known upper bound (RGap*), the number of DCs separated at integer points (nICut), the number of DCs separated at fractional points (nFCut), the number of cuts where at least one redundant disjunctions was removed (nRed), the time needed to solve the follower problem ($t_F$), the additional time needed to separate a DC ($t_S$), the number of instances solved to optimality and the total number of instances considered in each row (nSol).

The gaps are calculated as follows: RGap is calculated as $100 (z^*_R-LB_R)/z^*_R$, where $z^*_R$ and $LB_R$ are the best objective-function value and the lower bound at the end of the root node, respectively. In the $*$ counterparts of Gap and RGap, we use the best-known objective-function value of the instance over all the experiments described in this section, instead of $z^*$ and $z^*_R$. For the cutting-plane method, only a lower bound is available unless the instances is solved to optimality, thus we only provide Gap*.

In Table 1 each row presents average values over 10 instances with $n \in \{20,30,40,50\}$, and in Table 2, the instances are grouped according to the percentage of items that are controlled by the leader ($n_1/n \in \{0.5,0.75\}$). We observe that the removal of redundant disjunctions is particularly effective when fractional points are separated, and that for the cutting-plane method the redundant disjunctions are rarely detected. The latter can be explained by the fact that the problems solved to detect redundancy are more likely to be infeasible when considering local variable bounds. Moreover, we observe speed-ups of orders of magnitude when using G instead of using O. In terms of final gaps, for the most difficult instances (namely those from QBCov with $n = 50$ and all instances from QBMKP), the best method is BC-best, providing final average gaps which are two to 15 times lower than the respective gaps of the competing methods.

*Performance of the B &C on instances with two linking constraints.* We now turn our attention to the set of 140 binary instances with two linking constraints, 40 of them being from the benchmark set QBCov and 100 from QBMKP. For these instances, (CG-SOCP) has three disjunctions, one for the objective function, and one for each of the linking constraints. In order to have a fair comparison, out of 140 instances from this benchmark set, we only consider 123 instances for which at least one of the methods was either able to find a feasible solution, or to prove infeasibility (for 17 instances from QBMKP, the feasibility status remains unknown). We again compare the settings BC-best and BC-base and report the corresponding ECDFs in Fig. 7. Also here, significant improvements in the performance can be achieved thanks to a proper combination of separation and disjunction removal strategies (around 50% of instances are solved to optimality in the BC-base setting versus more than 80% when BC-best is used instead). More detailed results for these instances are provided in Tables 3 and 4. It is clear that under BC-best we are able to decrease the solution times for each group of instances and to detect many redundant disjunctions.

**Fig. 8:**
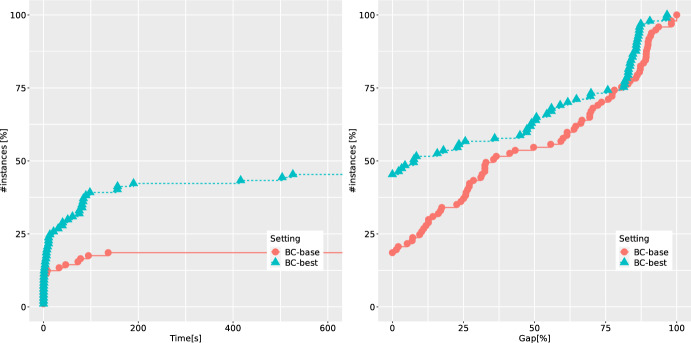
ECDFs reporting runtimes and final gaps for BC-base and BC-best over 97 integer instances with one linking constraint

*Performance of the B &C on instances with integer variables.* Finally, we also consider 100 additional instances from the benchmark set QBMKP with integer variables. For 97 instances (for which we were able to either find a feasible solution, or to prove infeasibility) the results comparing the settings BC-best and BC-base are summarized in Fig. 8. We observe that IBNPs with integer variables are more difficult for our method than their binary counterparts. The improvements obtained using the BC-best setting are still significant (we double the number of instances solved to optimality within the time limit), however, more than 50% of the instances from this benchmark set remain unsolved. These instances are the most difficult ones considered in this study, which can be explained by the much larger size of the search space. Based on the detailed results which are provided in Table 5, we observe that the number of explored branching nodes is orders of magnitude higher compared to the similar instances with binary variables, and the strength of DCs (in terms of the root bounds) is significantly weaker when integer variables are involved.

**Table 5:** Average results of BC-base and BC-best over 97 integer QBMKP instances with one linking constraint

$n_1/n$	Setting	*t*	Gap	Gap*	RGap	RGap*	nNode	nICut	nFCut	nRed	$t_F$	$t_S$	nSol
0.50	BC-base	517.6	64.6	53.3	96.0	55.7	43044.1	13409.9	–	–	411.0	98.6	7/47
	BC-best	464.6	45.8	43.9	95.9	49.5	36864.1	959.6	35208.5	13368.0	180.0	255.5	11/47
0.75	BC-base	471.1	25.9	21.6	86.4	23.8	123843.5	25398.6	–	–	246.5	204.2	11/50
	BC-best	258.4	21.4	10.2	84.3	19.2	18200.1	1990.8	15386.6	5604.7	97.0	148.9	33/50

## Conclusions and outlook

In this article, we demonstrated that SOCP-based DCs are an effective and promising methodology for solving a challenging family of discrete BPs with a convex quadratic objective and linear constraints in the follower problem. Although DCs have been employed with some success for several classes of MINLPs, their use and development for IBNPs is novel. The fact that we significantly outperform a state-of-the-art method for MIBLPs (after linearizing the nonlinear terms) indicates that further development of dedicated solution approaches for IBNPs exploiting nonlinear (and in particular SOCP-based) techniques is a promising endeavour.

There are still many open questions for future research. The proposed B &C could be enhanced by bilevel-specific preprocessing, or bilevel-specific valid inequalities (as this has been done for MIBLPs in e.g., [18, 19]). Problem-specific strengthening inequalities could be used within disjunctions to obtain stronger DCs, and finally outer-approximation could be used as an alternative to SOCP-based separation. It also remains open to study problem generalizations involving (discrete) follower problems with (multiple) conic constraints.

### Supplementary Information

Below is the link to the electronic supplementary material.

Supplementary file 1 (pdf 354 KB)

## Footnotes

1 This assumption is without loss of generality, as we can outer approximate second-order conic constraints of $\mathcal {P}$ and get an extreme optimal point by a simplex method.
